# Common pollen and related allergen components in patients with allergic diseases in the Beijing area

**DOI:** 10.3389/falgy.2024.1478392

**Published:** 2024-11-21

**Authors:** Yi-Bo Hou, Jin-Lu Sun

**Affiliations:** ^1^ State Key Laboratory of Complex Severe and Rare Diseases, Department of Allergy, Peking Union Medical College Hospital, Chinese Academy of Medical Sciences and Peking Union Medical College, Beijing, China; ^2^ Beijing Key Laboratory of Precision Medicine for Diagnosis and Treatment of Allergic Diseases, Allergy Department, National Clinical Research Center for Dermatologic and Immunologic Diseases, Peking Union Medical College Hospital, Chinese Academy of Medical Sciences and Peking Union Medical College, Beijing, China

**Keywords:** pollen, allergen components, allergic diseases, component-resolved diagnosis, CCD

## Abstract

**Background:**

Pollen is the most common outdoor allergen that causes allergic rhinitis and asthma, which seriously affects patient quality of life and extensive cross-reactivity occurs between pollen allergens.

**Methods:**

The study enrolled 84 patients with respiratory allergies and at least one pollen allergy who visited the clinic. Specific-IgE was detected via immunoblotting in the sera of patients with positive respiratory allergies to pollen. IgE of the components and cross-reactive carbohydrate determinants (CCD) were evaluated using a fluorescence-encoded microsphere assay.

**Results:**

Our results suggest that *Artemisia absinthium*, *Artemisia vulgaris, Humulus scandens*, *Amaranthus, Parietaria micrantha* allergies are most common in the northern region, and that weed pollen remains the major pollen allergen in the northern region. Among the different age groups, the positive rate of *Platanus* pollen allergens was significantly higher in patients ≤18 years of age than in those aged >18 years (55.56% vs. 9.17%, *χ*² = 0.55, *p* < 0.027). Patients with allergic rhinitis and asthma had an increased positive rate for *Betula* pollen allergen (20.00% vs. 37.93 *χ*² = 7.87, *p* = 0.005) and *Platanus* pollen allergen (27.27% vs. 51.72%, *χ*² = 11.05, *p* = 0.0008) than those with allergic rhinitis alone, although the allergen positivity rate did not significantly differ between sexes. In addition, the positivity of sIgE of allergen components did not reveal a correlation with clinical symptoms and anti-CCD IgE positivity was 1.19% (1/84) among all patients.

**Conclusion:**

The study found the distribution characteristics of common pollen allergens in Beijing among patients of different ages and genders and with different allergic diseases, as well as the relationship between pollen allergen components and symptoms. The positivity rate of CCD for respiratory allergic diseases in Beijing was not high as well.

## 1 Introduction

Allergic diseases usually involve pathological changes, such as dysfunction or tissue damage, caused by continuous stimulation of the organism with specific allergens or restimulation with the same allergen (1). The incidence of allergic diseases has been increasing annually, and approximately one-third of children in developed countries suffer from at least one allergic disease that seriously affects their lives (2).

Allergens (outdoor, indoor, and occupational allergens, etc) are the main causes of allergic diseases and can induce allergic rhinitis, dermatitis, asthma, and pollen-food allergy syndrome (3). Pollen is the main outdoor allergen that causes seasonal allergic diseases and affects human health worldwide. The distribution of pollen allergens varies regionally, owing to differences in geography and climate. In Europe, >11% of the population has a pollen allergy, which primarily manifests as allergic rhinitis or asthma (4). In China, *Artemisia*, *Chenopodium*, and *Humulus* are the most common pollen types among patients with pollen-induced allergic rhinitis, with a prevalence rate of 18.5% for pollen-related allergic rhinitis (5), and Artemisia pollen exhibits the highest association among major pollen species contributing to seasonal allergic asthma (6). The prevalence of artemisia pollen allergy in Europe ranges from 10%–14% (7) while the prevalence in the grassland area of Inner Mongolia in China is 14.38% (8), although some high-prevalence areas, this can reach >50% (9). In addition, the most common allergen in southern and eastern China is the house dust mite while pollen sensitization is high in central, northern, and northwestern China (10, 11).

Different pollen allergens share the same sensitization phenomenon, mainly owing to allergen cross-reactivity. Allergen cross-reactivity refers to allergens from different pollens inducing the same antigen–antibody reaction, which consequently induces similar allergic symptoms. In addition, a specific class of cross-reactivity is caused by sugar chains on allergen molecules, typically cross-reactive carbohydrate (CCDs) and *α*-Gal antigenic determinants. CCDs are widely found in glycoproteins of plants and animals (e.g., pollen, bees, soybeans, peanuts, epithelium of cats and dogs, acorns) and can induce the production of CCD-specific IgE antibodies that cross-react with various allergens, whose biological significance is still unknown (12). *α*-Gal antigenic determinants are present on galactose-alpha-1,3-galactose, which is the main hematological molecule on erythrocytes of nonprimate mammals, such as pigs, cattle, sheep, cats, and dogs. Induced IgE can be produced by CCDs, the main blood-grouping substance in red blood cells. The induced production of IgE can cause severe delayed-type allergic reactions (13). CCD-induced sIgE is disruptive for screening pollen allergens. A Chinese study of the sensitization pattern of serum samples from patients with allergic diseases found that the pollen CCD positivity rate reached 39.4%, and the analysis found that CCD was associated with sensitization rows of ambrosia artemisiifolia and quinoa pollen (14). Therefore, the influence of CCD-IgE should be considered when a mismatch is present between serological test results and clinical manifestations.

With the rapid development of molecular technology in recent years, allergen-component proteins have been discovered and characterized. Allergen component-resolved diagnosis, also known as component-resolved diagnosis (CRD), has received widespread attention as a new method for detecting allergen component–specific IgE. CRD uses natural or recombinant single-component allergens, rather than crude extracts of allergens, to detect sIgE, and can avoid nonallergenic interference in crude extracts to detect allergenic substances in these extracts (15). CRD contributes to the precise diagnosis of allergic diseases and may provide a basis for the precise treatment of allergic diseases.

In this study, we used fluorescently labelled microspheres in enzyme-linked immunosorbent assay format to detect allergen-specific IgE and focus on the epidemiology of pollen allergens in allergic diseases in the northern region of China.

## 2 Materials and methods

### 2.1 Study population

This study was approved by the Medical Ethics Committee of the Peking Union Medical College Hospital (JS-3353). Sera was collected from 84 patients who visited the Department of Metabolic Reactions of Peking Union Medical College Hospital between January 2023 and December 2023 and used to study CCD adsorbents in the diagnostic testing of sera components from patients with hay fever.

### 2.2 Patient inclusion criteria

The inclusion criteria were a history of pollen allergy and respiratory symptoms after exposure to pollen; serum sIgE to pollen from one of *Cupressus funebris*, *Platanus, Betula*, *Ambrosia artemisiifolia*, *Humulus scandens*, and *Artemisia vulgaris*>0.35KUA/L(ImmunoCAP system, Thermo-Fisher, Sweden) was accepted as sufficient and can be combined with other pollen allergies, although the dust mite and fungal sIgE was <0.35KUA/L. Exclusion criteria were recent omalizumab injections for symptom control, immunodeficiencies, or autoimmune diseases, and an inability for blood sample collection due to severe allergic reactions.

### 2.3 Hand collection and preservation of serum samples

In total, 2 ml of patient venous blood was collected, allowed to stand for 2 h at room temperature, and then centrifuged 3,000 rpm at 4℃ for 10 min. The upper fraction was removed and divided into 300-ml aliquots for storage at −80℃.

### 2.4 Detection of IgE to tree and grass pollen allergens

The sIgE concentration of pollen allergens in sera of patients with allergic diseases was detected using an allergen-specific IgE detection kit (Immunoblotting Method) from Hangzhou Zheda Dixun Biological Gene Engineering Co. The s-IgE of allergic components were also detected using allergen-component specific IgE detection from Hangzhou Zheda Dixun Biological Gene Engineering Co.

### 2.5 Statistical analysis

Data were processed using GraphPad Prism and SPSS23.0 statistical software. Normal information was expressed as mean ± standard deviation while non-normal measurements were expressed as median (interquartile range), and counts were expressed as percentages. Comparisons between groups were made using chi-square test or Fisher's exact probability method; a *p*-value < 0.05 was considered different.

## 3 Results

### 3.1 Patient demographic characteristics

Eighty-four eligible patients were enrolled in this study, including 42 males and 42 females, with a mean age of 24.78. Patients were divided into two groups according to their age: 48 patients were ≤18 years old and 36 patients were >18 years old. Patients were primarily from Beijing, China. In total, 55 patients had allergic rhinitis while 29 had allergic rhinitis and asthma. Demographic characteristics of the patients are presented in Table 1.

**Table 1:** Demographic characteristics of enrolled patients.

Characteristic	Total
	*N* = 84
Age, mean ± SD	24.78 ± 15.11
Gender, *n* (%)	
Male	42 (50%)
Female	42 (40%)
Age, *n* (%)	
≤18	36 (41.67%)
>18	48 (58.3%)
Diagnosis, *n* (%)	
Allergic rhinitis	55 (59.52%)
Allergic rhinitis with asthma	29 (40.48%)

### 3.2 Distribution of pollen allergen sIgE in patients

**Figure 1:**
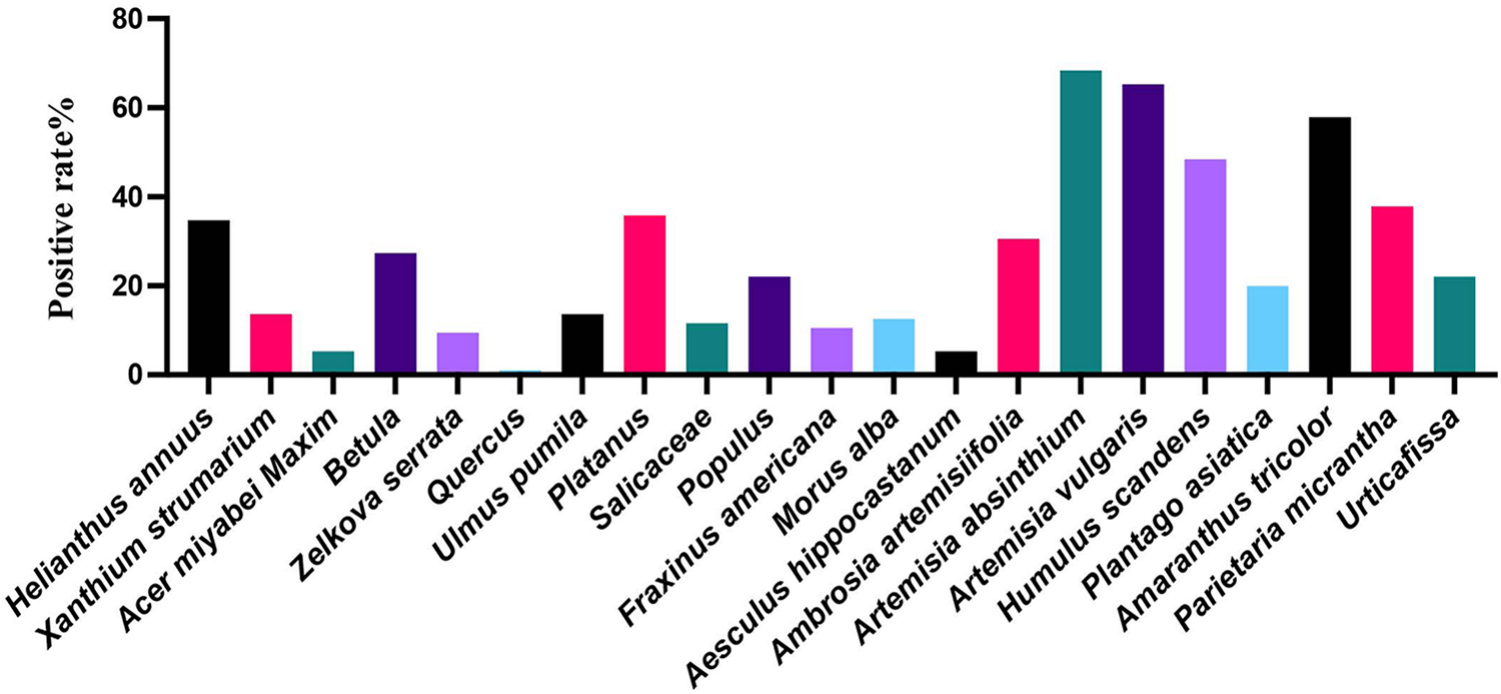
Positivity rates for pollen allergens in sIgE.

Of the 84 patients enrolled, 33 (34.74%) were allergic to *Helianthus annuus*, 13 (13.68%) to *Xanthium strumarium*, 5 (5.26%) to *Acer miyabei Maxim*, 26 (27.37%) to *Betula*, 9 (9.47%) to *Zelkova serrata*, 1 (1.05%) to *Quercus*, 13 (13.68%) to *Ulmus pumila*, 34 (35.79%) to *Platanus*, 11 (11.58%) allergic to *Salicaceae*, 21 (22.11%) allergic to *Populus*, 10 (10.53%) allergic to *Fraxinus americana*, 12 (12.63%) allergic to *Morus alba*, 5 (5.26%) allergic to *Aesculus hippocastanum*, 29 (30.53%) allergic to *Ambrosia artemisiifolia*, 65 (68.42%) allergic to *Artemisia absinthium*, 62 (65.26%) allergic to *Artemisia vulgaris*, 46 (48.42%) to *Humulus scandens*, 19 (20.00%) to *Plantago asiatica*, 55 (57.89%) to *Amaranthus tricolor*, 36 (37.89%) to *Parietaria micrantha*, and 21 (22.11%) to *Urticafissa* (Figure 1). Our results suggest that allergies to *Artemisia absinthium*, *Artemisia vulgaris*, *Humulus scandens*, *Amaranthus tricolor*, and *Parietaria micrantha* are most common in the northern region and that weed pollen remains the major pollen allergen in the northern region.

### 3.3 Distribution of pollen allergen positivity in different age groups and gender groups

The results of pollen allergen sIgE testing in different age groups showed that the positivity rate of *Platanus* pollen allergen was significantly higher in patients ≤18 years old than in those >18 years old (55.56% vs. 9.17%, *χ*^2^ = 0.55, *p* < 0.027), which indicates that the positivity rate of pollen sIgE gradually decreases with age (Table 2). No significant difference was found in the positivity rate of the other pollen types between the two age groups (*p* > 0.05). We analyzed the pollen allergen positivity rates with regard to patient gender and found no significant differences in the allergen positivity rates between genders (all *p*-values were >0.05) (Supplementary Table S1).

**Table 2:** Distribution of allergens among different age groups.

Allergen	*n* (%)		*χ* ^2^	*p*
	≤18	>18		
*Helianthus annuus*	12 (33.33%)	21 (43.75%)	0.55	0.458
*Xanthium strumarium*	5 (13.89%)	8 (16.67%)	0.00	0.965
*Acer miyabei* Maxim	3 (8.33%)	2 (4.17%)	0.11	0.739
*Betula*	7 (19.44%)	19 (39.58%)	3.02	0.082
*Zelkova serrata*	4 (11.11%)	5 (10.42%)	0.00	1.000
*Quercus*	12.78%)	0 (0.00%)	0.02	0.885
*Ulmus pumila*	7 (19.44%)	7 (14.58%)	0.09	0.767
*Platanus*	20 (55.56%)	14 (29.17%)	4.90	0.027
*Salicaceae*	5 (13.89%)	6 (12.50%)	0.00	1.000
Populus	10 (27.78%)	11 (22.92%)	0.06	0.799
*Fraxinus americana*	6 (16.67%)	4 (8.33%)	0.68	0.408
*Morus alba*	6 (16.67%)	6 (12.50%)	0.05	0.822
*Aesculus hippocastanum*	3 (8.33%)	2 (4.17%)	0.11	0.739
*Ambrosia artemisiifolia*	11 (30.56%)	18 (37.50%)	0.19	0.667
*Artemisia absinthium*	25 (69.44%)	40 (83.33%)	1.54	0.214
*Artemisia vulgaris*	25 (69.44%)	37 (77.08%)	0.29	0.591
*Humulus scandens*	21 (58.33%)	25 (52.08%)	0.12	0.728
*Plantago asiatica*	9 (25.00%)	10 (20.83%)	0.04	0.851
*Amaranthus tricolor*	24 (66.67%)	31 (64.58%)	0.00	1.000
*Parietaria micrantha*	16 (44.44%)	20 (41.67%)	0.00	0.975
*Urticafissa*	11 (30.56%)	10 (20.83%)	0.58	0.445

### 3.4 Positive distribution of pollen allergens in different diseases group

Our results showed that patients with allergic rhinitis and allergic rhinitis combined with asthma had been predominantly exposed to pollen of *Helianthus annuus*, *Betula*, *Platanus*, *Salicaceae*, *Ambrosia artemisiifolia, Artemisia absinthium, Artemisia vulgaris, Humulus scandens*, *Amaranthus tricolor, Parietaria micrantha*. *Betula* (20.00%: 37.93%, *χ*^2^ = 7.87, *p* = 0.005) and *platanus* pollen allergen positivity (27.27%: 51.72%, *χ*^2^ = 11.05, *p* = 0.0008) in patients with allergic rhinitis and asthma were significantly higher than those in the allergic rhinitis group. The remaining pollen allergen positivity rates did not significantly differ between the two disease groups (Table 3).

**Table 3:** Distribution of allergen positivity between different disease groups.

Allergen	*n* (%)		*χ* ^2^	*p*
	Allergic rhinitis	Allergic rhinitis and asthma		
*Helianthus annuus*	20 (36.36%)	9 (31.03%)	0.03	0.857
*Xanthium strumarium*	9 (16.36%)	2 (6.90%)	1.37	0.239
*Acer miyabei* Maxim	2 (3.64%)	3 (10.34%)	2.77	0.095
*Betula*	11 (20.00%)	11 (37.93%)	7.87	0.005
*Zelkova serrata*	4 (7.27%)	4 (13.79%)	0.10	0.751
*Quercus*	0 (0.00%)	1 (3.45%)	2.19	0.138
*Ulmus pumila*	6 (10.91%)	5 (17.24%)	0.46	0.494
*Platanus*	15 (27.27%)	15 (51.72%)	11.05	0.0008
*Salicaceae*	4 (7.27%)	5 (17.24%)	0.78	0.374
Populus	9 (16.36%)	9 (31.03%)	1.35	0.244
*Fraxinus americana*	5 (9.09%)	5 (17.24%)	2.44	0.118
*Morus alba*	6 (10.91%)	5 (17.24%)	0.00	0.961
*Aesculus hippocastanum*	2 (3.64%)	3 (10.34%	1.48	0.223
*Ambrosia artemisiifolia*	16 (29.09%)	9 (31.03%)	0.14	0.703
*Artemisia absinthium*	36 (65.45%)	23 (79.31%)	1.45	0.227
*Artemisia vulgaris*	34 (61.82%)	22 (75.86%)	1.25	0.261
*Humulus scandens*	23 (41.82%)	17 (58.62%)	4.36	0.036
*Plantago asiatica*	10 (18.18%)	8 (27.59%)	1.30	0.253
*Amaranthus tricolor*	34 (61.82%)	14 (48.28%)	2.00	0.156
*Parietaria micrantha*	23 (41.82%)	9 (31.03%)	0.39	0.527
*Urticafissa*	12 (21.82%)	7 (24.14%)	0.00	0.927

### 3.5 Positivity of different allergen components and the relationship between sIgE of components and different diseases

**Figure 2:**
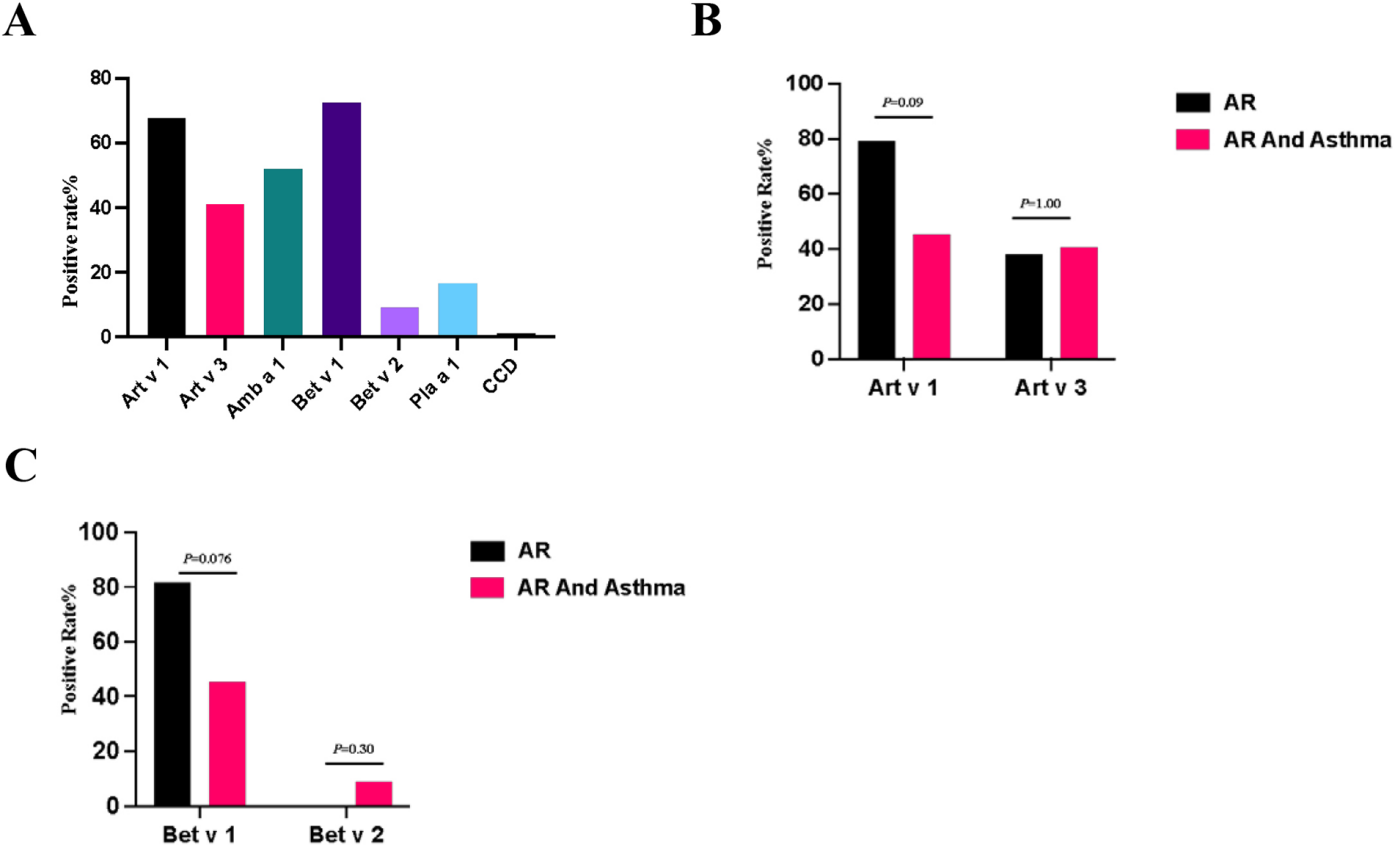
**(A)** Positive rate of pollen components sIgE. **(B)** Positive rate of Art v 1 and Art v 3 within different disease groups. **(C)** Positive rate of Bet v 1 and Bet v 2 within different disease groups. Compared by chi-square test, the positive rate of each whole disease group was analyzed.

For the 84 patients with pollen allergies, the positivity rate of Art v 1 in artemisia sIgE–positive patients was 67.86% (38/56), Art v 3 was 41.07% (23/56), Amb a 1 in dwarf ambrosia artemisiifolia sIgE–positive was 52% (13/52), Bet v 1 was 72.73% (16/22) and Bet v 2 was 9.09% (2/22) in birch sIgE–positive patients, Pla a 1 in *platanus* sIgE–positive patients was positive in 16.67% (5/30), and anti-CCD-IgE was positive in 1.19% (1/84) of all patients (Figure 2A).The positive rate of sIgE of Art v 1 was not significantly different in patients with different airway symptoms (*p* = 0.09) and Art v 3 did not show any statistically significant differences either (*p* = 1.00) (Figure 2B). The positivity of sIgE of Bet v 1 and Bet v 2 was not different significantly between different airway diseases (Figure 2C).

## 4 Discussion

With rapid urbanization in China, changes in vegetation types due to urban greening have affected the characteristics of pollen allergies in various regions. A wide range of pollen-producing species are abundant in northern China, and pollen-induced allergic diseases are having an increasing impact on people. In this study, we included 84 patients with pollen allergy whose symptoms manifested as respiratory diseases. First, we analyzed pollen allergen sensitization in patients with respiratory allergic diseases in Beijing and showed that patients with allergic rhinitis and those with allergic rhinitis combined with asthma in the northern region of China were mainly allergic to tree and weed pollens, of which *Platanus*, birch, and *Salicaceae* pollen were the major tree pollen allergens, and *Artemisia absinthium, Artemisia vulgaris, Humulus scandens, Amaranthus tricolor, Parietaria micrantha* and *Helianthus annuus* were the major weed pollens causing hay fever, which dominate in the development of allergic diseases.

*Platanus* is widely planted as an urban tree in many cities, including Beijing, and is considered to be one of the main pollen allergens causing spring hay fever (16). Our results showed that *Platanus* pollen is one of the major tree pollen sources of respiratory allergy, and the positive rate of *Platanus* pollen allergy was higher in patients with allergic rhinitis combined with asthma than in those with allergic rhinitis alone, which may be related to the course of the pollen allergy. In addition, our results showed a higher positive rate for plane tree pollen in the immature group, which was significantly different from that in the adult group, suggesting that minors may be more sensitive to *Platanus* pollen allergens.

In the present study, the positive rate of birch pollen allergen was second only to that of *Platanus*. Birch pollen is a common allergen causing allergic rhinitis in spring, which is mainly distributed in the central and northern regions of China, and the positive rate of birch pollen sensitization ranged from 7% to 25% in a cross-sectional study (17, 18). Birch pollen is the most important allergenic pollen in Europe and the positive rate of the birch pollen skin prick test in Switzerland is up to 54% (19). In addition, *Salicaceae* is a major silvicultural species for protection forests and saprophytic forests and is a common tree species for urban greening in China. Our study also showed that pollen plays a major role in pollen allergies. *Salicaceae* is a common tree species or urban greening in China, being planted widely in many regions, and *Salicaceae* is the principal afforestation species for protection forests and Suzheng productive forests. Our study found that *Salicaceae* pollen was one of the major pollen allergens in the northern region of China (20).

Artemisia pollen is the main allergen causing hay fever in summer and autumn in China because of the wide distribution of vegetation, large pollen volume, and strong allergenicity, which seriously affects human health (21). Our study found a positive rate of Artemisia allergy as high as 65.26% in patients with respiratory symptoms, which may indicate that with the cultivation of Artemisia plants, Artemisia pollen allergy will become more serious in the northern part of China. *Humulus* pollen, which has been linked to adverse effects on human health, is also the most prevalent pollen species during the summer and autumn months in the northern regions of the country. Our study revealed that the positive rate of *Humulus* pollen allergy was 48.42%. Furthermore, the presence of certain unidentified pollens is also affecting patients with pollen allergies during the autumn season. These include *Amaranthus* pollen, which has been observed from May to August. Our findings indicate that the high positivity rate of this pollen may be a contributing factor to the prevalence of pollen allergies during the summer months. Wallflower pollen, a prevalent allergen in the Mediterranean region and a significant contributor to the prevalence of hay fever in European countries such as Italy and Spain, has been inadequately studied in China (22, 23). Our results showed a high positive rate of wallflower pollen allergy, suggesting that this may be one of the pollens responsible for allergic diseases in the Beijing region.

We performed serum IgE assays for the major allergenic components of several pollens. Art v 1, the major allergenic component of *Artemisia vulgaris*, belongs to the family of defensin-like proteins, which are usually associated with natural immunity in plants and cause a strong inflammatory response in allergic reactions (24). Art v 3 belongs to the nonspecific lipid transfer protein type 1 (nsLTP) family, a cross-allergen that can cause food allergies associated with peaches (25). Our results showed that positivity rate of Art v 1 was high in patients allergic to artemisia species pollen, whereas that of Art v 3 was lower, but they did not show statistically significant difference in patients with different airway symptoms. Amb a 1 is the major allergenic component of ambrosia artemisiifolia pollen, and although our results showed that the positivity rates of ambrosia artemisiifolia pollen and of Amb a 1 were lower than that of artemisia species pollen, the clinical symptoms may be caused by lower ambrosia artemisiifolia pollen concentration, indicating that symptoms may have lower thresholds (26). Clinical symptoms may be more severe in patients with ambrosia *Artemisiifolia* pollen allergy because of the high ambrosia *Artemisiifolia* pollen concentration and long pollination season (27). But our results showed no significant difference in ambrosia *Artemisiifolia* positivity between the allergic rhinitis and allergic rhinitis combined with asthma groups. Pla a 1, the main allergen of *Platanus* pollen, is a putative convertase inhibitor, and we detected a low positive rate of sensitization to Pla a1, which differs from reported results. However, our results showed that *Platanus* pollen allergy was positive in 35.71% of patients. These results may be related to our detection method, which therefore needs to be changed, or one or more samples must be collected for further validation. The birch pollen allergen component Bet v 1 has cross-reactivity and can cause pollen-food allergy syndrome while another study found that 82.8% of patients with pollen-food allergy syndrome tested positive for Bet v 1 (28). In different European countries, the Bet v 1–positivity rate in birch pollen allergic patients ranges from 62% to 100%, depending on the region (29, 30). Bet v 2 is a member of the pan-allergen family of profilins. As one of the major allergenic components of birch pollen, Bet v 2 has extensive cross-reactivity with allergens in the profilin family of other pollen and food sources, such as *Ambrosia artemisiifolia*, and has been found to be a cross-reactivating agent in a wide range of food sources, including *Ambrosia artemisiifolia* (Amb a 8), *Artemisia vulgaris* (Art v 4), oranges (Cit s 2), and melons (Cuc m 2) (31, 32). In addition, the levels of sIgE positivity for Bet v 1 and Bet v 2 did not show a significant difference across the various airway diseases examined in this study.

Finally, we tested CCD-sIgE and found that only 1 out of 84 patients showed a positive result, which is different from the outcomes of previous studies. To ascertain the veracity of this outcome, we proceeded to test CCD-sIgE in 41 patients with pollen allergies and the results showed that all patients had CCD-sIgE test levels below 0.35 IU/ml (Supplementary Figure S1). However, a study of pollen sensitization patterns in southern China found a CCD positivity rate of 39.4% (14), which we hypothesize may be related to the pollen species present in different regions. The results indicate that the positivity rate of CCD-sIgE in pollen-sensitized patients in northern China is markedly low, and its utility in pollen-sensitized patients requires further validation.

## 5 Conclusions

Our study found that weed pollens and tree pollens are the main causes of airway allergic diseases, *Platanus* pollens appear to predominantly affect minors, while birch and *Platanus* pollens may severely impact both the upper and lower airways. Additionally, there was no significant correlation between different pollen types and gender. Our study did not reveal a correlation between allergen components and clinical symptoms, suggesting that further investigation with a larger sample size may be necessary. Furthermore, the prevalence of positive CCD-sIgE results in patients with respiratory allergic diseases in Beijing is relatively low. Additional research is required to confirm the clinical diagnostic value of anti-CCD sIgE.

## Data Availability

The raw data supporting the conclusions of this article will be made available by the authors, without undue reservation.

## References

[1] Cardona V Ansotegui IJ Ebisawa M El-Gamal Y Fernandez Rivas M Fineman S World allergy organization anaphylaxis guidance 2020. World Allergy Organ J. (2020) 13(10):100472. 10.1016/j.waojou.2020.100472 33204386 PMC7607509

[2] Custovic A. To what extent is allergen exposure a risk factor for the development of allergic disease? Clin Exp Allergy. (2015) 45(1):54–62. 10.1111/cea.12450 25381695

[3] Demoly P Tanno LK Akdis CA Lau S Calderon MA Santos AF Global classification and coding of hypersensitivity diseases - an EAACI - WAO survey, strategic paper and review. Allergy. (2014) 69(5):559–70. 10.1111/all.12386 24650345

[4] Altmann F. Coping with cross-reactive carbohydrate determinants in allergy diagnosis. Allergo J Int. (2016) 25(4):98–105. 10.1007/s40629-016-0115-3 27656353 PMC5016538

[5] Wang XY Ma TT Wang XY Zhuang Y Wang XD Ning HY Prevalence of pollen-induced allergic rhinitis with high pollen exposure in grasslands of northern China. Allergy. (2018) 73:1232–43. 10.1111/all.13388 29322523 PMC6033040

[6] Gao Z Fu WY Sun Y Gao B Wang HY Liu M Artemisia pollen allergy in China: component-resolved diagnosis reveals allergic asthma patients have significant multiple allergen sensitization. Allergy. (2019) 74(2):284–93. 10.1111/all.13597 30155917 PMC6587742

[7] Schülke S Kuttich K Wolfheimer S Duschek N Wangorsch A Reuter A Conjugation of wildtype and hypoallergenic mugwort allergen art v 1 to flagellin induces IL-10-DC and suppresses allergen-specific TH2-responses *in vivo*. Sci Rep. (2017) 7(1):11782. 10.1038/s41598-017-11972-w 28924222 PMC5603567

[8] Ma T Zhuang Y Wang H Wei Q Shi H Ning H Analysis of sensitization characteristics of artemisia pollen in the Inner Mongolian grassland region of China. Lin Chuang Er Bi Yan Hou Tou Jing Wai Ke Za Zhi. (2020) 34(12):1092–6. 10.13201/j.issn.2096-7993.2020.12.009 33254342 PMC10127775

[9] Jing WU Lan S. Investigation on airborne allergenic pollen and analysis on clinical data of pollinosis in Hohhot. Occupation and Health. (2013) 29(03):266–9. 10.13329/j.cnki.zyyjk.2013.03.043

[10] Li CY Liu XJ Xu HX Fu Q Xu DY Cui XB Analysis of pollen sensitization characteristics of artemisia allergic rhinitis in three urban and rural areas of Inner Mongolia. Zhonghua Yu Fang Yi Xue Za Zhi [Chinese Journal of Preventive Medicine]. (2024) 58(6):806–14. 10.3760/cma.j.cn112150-20231109-00324 38955727

[11] Lou H Ma S Zhao Y Cao F He F Liu Z Sensitization patterns and minimum screening panels for aeroallergens in self-reported allergic rhinitis in China. Sci Rep. (2017) 7(1):9286. 10.1038/s41598-017-10111-9 28839248 PMC5570894

[12] Vieths S Scheurer S Ballmer-Weber B. Current understanding of cross-reactivity of food allergens and pollen. Ann N Y Acad Sci. (2002) 964:47–68. 10.1111/j.1749-6632.2002.tb04132.x 12023194

[13] Commins SP Satinover SM Hosen J Mozena J Borish L Lewis BD Delayed anaphylaxis, angioedema, or urticaria after consumption of red meat in patients with IgE antibodies specific for galactose-alpha-1,3-galactose. J Allergy Clin Immunol. (2009) 123(2):426–33. 10.1016/j.jaci.2008.10.052 19070355 PMC3324851

[14] Xu L Luo W Lu Y Huang Z Yu X Liao C A comprehensive analysis of the components of common weed pollen and related allergens in patients with allergic diseases in southern China. Mol Immunol. (2022) 147:180–6. 10.1016/j.molimm.2022.05.005 35633613

[15] Matricardi PM Kleine-Tebbe J Hoffmann HJ Valenta R Hilger C Hofmaier S EAACI Molecular allergology user’s guide. Pediatr Allergy Immunol. (2016) 27(Suppl 23):1–250. 10.1111/pai.12563 27288833

[16] Wang XY Tian ZM Ning HY. Association between airborne pollen distribution and allergic diseases in Beijing urban area. Lin Chuang Er Bi Yan Hou Ke Za Zhi. (2017) 31(10):757–61. 10.13201/j.issn.1001-1781.2017.10.005 29771037

[17] Li JD Du ZR Liu J Xu YY Wang RQ Yin J. Characteristics of pollen-related food allergy based on individual pollen allergy profiles in the Chinese population. World Allergy Organ J. (2020) 13(5):100120. 10.1016/j.waojou.2020.100120 32435327 PMC7229292

[18] Xu M Ye Q Zhang J Huang Z Wang Y Liu J Study on sIgE distribution characteristics and the sensitization pattern of allergen in 1 161 patients with allergic diseases of respiratory tract in northwest China. Zhonghua Yu Fang Yi Xue Za Zhi [Chinese Journal of Preventive Medicine]. (2023) 57(9):1355–63. 10.3760/cma.j.cn112150-20230507-00352 37743295

[19] D'Amato G Spieksma FT Liccardi G Jäger S Russo M Kontou-Fili K Pollen-related allergy in Europe. Allergy. (1998) 53(6):567–78. 10.1111/j.1398-9995.1998.tb03932.x 9689338

[20] Cheng S Yu Y Ruan B. Species and distribution of airborne pollen plants in major cities of China. Chin J Allergy Clin Immunol. (2015) 2015:136–41. 10.3969/j.issn.1673-8705.2015.02.011

[21] Li C Huang H Liu X Liu X. Research progress on the characteristics of artemisia pollen allergens and related pollinosis. Zhonghua Yu Fang Yi Xue Za Zhi [Chinese Journal of Preventive Medicine]. (2022) 56(6):748–54. 10.3760/cma.j.cn112150-20220314-00232 35785856

[22] D'Amato G Ruffilli A Sacerdoti G Bonini S. Parietaria pollinosis: a review. Allergy. (1992) 47(5):443–9. 10.1111/j.1398-9995.1992.tb00661.x 1485645

[23] D'Amato G Cecchi L Bonini S Nunes C Annesi-Maesano I Behrendt H Allergenic pollen and pollen allergy in Europe. Allergy. (2007) 62(9):976–90. 10.1111/j.1398-9995.2007.01393.x 17521313

[24] Himly M Jahn-Schmid B Dedic A Kelemen P Wopfner N Altmann F Art v 1, the major allergen of mugwort pollen, is a modular glycoprotein with a defensin-like and a hydroxyproline-rich domain. FASEB J. (2003) 17(1):106–8. 10.1096/fj.02-0472fje 12475905

[25] Pastorello EA Pravettoni V Farioli L Rivolta F Conti A Ispano M Hypersensitivity to mugwort (*artemisia vulgaris*) in patients with peach allergy is due to a common lipid transfer protein allergen and is often without clinical expression. J Allergy Clin Immunol. (2002) 110(2):310–7. 10.1067/mai.2002.125830 12170274

[26] Weber RW. Patterns of pollen cross-allergenicity. J Allergy Clin Immunol. (2003) 112(2):229–39.; quiz 40. 10.1067/mai.2003.1683 12897724

[27] Biedermann T Kuna P Panzner P Valovirta E Andersson M de Blay F The SQ tree SLIT-tablet is highly effective and well tolerated: results from a randomized, double-blind, placebo-controlled phase III trial. J Allergy Clin Immunol. (2019) 143(3):1058–66.e6. 10.1016/j.jaci.2018.12.1001 30654054

[28] Wang X Chen L Ding J Wang H Wang X. Profiles of birch allergen component sensitization and its association with pollen food allergy syndrome in Northern China. J Asthma Allergy. (2023) 16:1241–50. 10.2147/JAA.S427764 38022747 PMC10656847

[29] Ciprandi G Comite P Mussap M De Amici M Quaglini S Barocci F Profiles of birch sensitization (bet v 1, bet v 2, and bet v 4) and oral allergy syndrome across Italy. J Investig Allergol Clin Immunol. (2016) 26(4):244–8. 10.18176/jiaci.0041 27470643

[30] Movérare R Westritschnig K Svensson M Hayek B Bende M Pauli G Different IgE reactivity profiles in birch pollen-sensitive patients from six European populations revealed by recombinant allergens: an imprint of local sensitization. Int Arch Allergy Appl Immunol. (2002) 128(4):325–35. 10.1159/000063855 12218371

[31] Högerle C San Nicolo M Gellrich D Eder K Gröger M. Clinical relevance of profilin sensitization concerning oral allergy syndrome in birch pollen sensitized patients. J Asthma Allergy. (2022) 15:249–55. 10.2147/JAA.S348650 35221697 PMC8866351

[32] Ebo DG Hagendorens MM Bridts CH De Clerck LS Stevens WJ. Sensitization to cross-reactive carbohydrate determinants and the ubiquitous protein profilin: mimickers of allergy. Clin Exp Allergy. (2004) 34(1):137–44. 10.1111/j.1365-2222.2004.01837.x 14720274

