# Supplementary material for: Common pollen and related allergen components in patients with allergic diseases in the Beijing area
Source: Front Allergy. 2024 Nov 21;5:1478392. doi: 10.3389/falgy.2024.1478392 (PMC11617527; doi:10.3389/falgy.2024.1478392)

Table 1 Distribution of allergen positivity among different gender groups

| Allergen | n (%) | | χ² | *p* |
| --- | --- | --- | --- | --- |
|  | Male | Female |  |  |
| *Helianthus annuus* | 14 (33.33%) | 15 (35.71%) | 0.000 | 1.000 |
| *Xanthium strumarium* | 6 (14.29%) | 5 (11.90%) | 0.000 | 1.000 |
| *Acer miyabei* Maxim | 3 (7.14%) | 2 (4.76%) | 0.000 | 1.000 |
| *Betula* | 13 (30.95%) | 9 (21.43%) | 0.554 | 0.457 |
| *Zelkova serrata* | 4 (9.52%) | 4 (9.52%) | 0.000 | 1.000 |
| *Quercus* | 1 (2.38%) | 0 (0.00%) | 0.000 | 1.000 |
| *Ulmus pumila* | 7 (16.67%) | 4 (9.52%) | 0.418 | 0.518 |
| *Platanus* | 19 (45.24%) | 11 (26.19%) | 2.541 | 0.111 |
| *Salicaceae* | 7 (16.67%) | 2 (4.76%) | 1.991 | 0.158 |
| Populus | 11 (26.19%) | 7 (16.67%) | 0.636 | 0.425 |
| *Fraxinus americana* | 7 (16.67%) | 3 (7.14%) | 1.022 | 0.312 |
| *Morus alba* | 8 (19.05%) | 3 (7.14%) | 1.674 | 0.196 |
| *Aesculus hippocastanum* | 3 (7.14%) | 2 (4.76%) | 0.000 | 1.000 |
| *Ambrosia artemisiifolia* | 12 (28.57%) | 13 (30.95%) | 0.000 | 1.000 |
| *Artemisia absinthium* | 31 (73.81%) | 28 (66.67%) | 0.228 | 0.633 |
| *Artemisia vulgaris* | 29 (69.05%) | 27 (64.29%) | 0.054 | 0.817 |
| *Humulus scandens* | 24 (57.14%) | 16 (38.10%) | 2.339 | 0.126 |
| *Plantago asiatica* | 12 (28.57%) | 6 (14.29%) | 1.768 | 0.184 |
| *Amaranthus tricolor* | 25 (59.52%) | 23 (54.76%) | 0.049 | 0.825 |
| *Parietaria micrantha* | 18 (42.86%) | 14 (33.33%) | 0.454 | 0.500 |
| *Urticafissa* | 13 (30.95%) | 6 (14.29%) | 2.449 | 0.118 |

Figure 1 Levels of CCD specific IgE in 41 samples


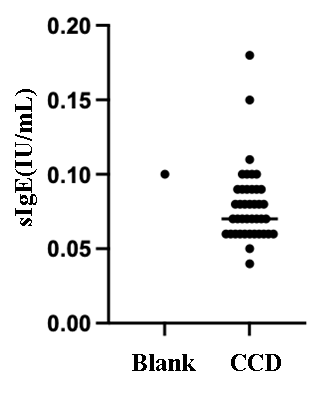

Supplement: Supplementary file 1 [file Datasheet1.docx]
